# A straightforward chemobiocatalytic route for one-pot valorization of glucose into 2,5-bis(hydroxymethyl)furan

**DOI:** 10.1186/s40643-024-00758-4

**Published:** 2024-04-18

**Authors:** Xuan-Ping Liao, Qian Wu, Min-Hua Zong, Ning Li

**Affiliations:** School of Food Science and Engineering, South China University of Technology, 381 Wushan Road, Guangzhou, 510640 China

**Keywords:** Alcohol dehydrogenases, Biomass conversion, Biobased chemicals, Chemobiocatalysis, Furans

## Abstract

Direct conversion of inexpensive biomass into value-added chemicals via furanic platform molecules is highly attractive. In this work, we present a straightforward chemobiocatalytic route for glucose valorization into 2,5-bis(hydroxymethyl)furan (BHMF) in one pot, with no purification of the intermediate 5-hydroxymethylfurfural (HMF). Six candidate alcohol dehydrogenase (ADH) genes were located from *Meyerozyma guilliermondii* SC1103, based on comparative transcriptome analysis and real-time quantitative polymerase chain reaction. An ADH (*Mg*ADH1) was identified upon evaluation of catalytic performances of recombinant *Saccharomyces cerevisiae* harboring candidate ADHs in HMF reduction. Soluble expression of the enzyme in *S. cerevisiae* was greatly enhanced by its codon optimization, leading to improved HMF tolerance (up to 400 mM). In a fed-batch process, the desired product of approximately 473 mM (60.5 g/L) was produced within 30 h by recombinant *S. cerevisiae*_*Mg*ADH1. A chemobiocatalytic route toward BHMF was constructed by merging CaCl_2_-mediated isomerization and dehydration with biocatalytic reduction with an overall yield of approximately 42%, starting from glucose. This work may pave the way for green manufacture of valuable biobased chemicals.

**Supplementary Information:**

The online version contains supplementary material available at 10.1186/s40643-024-00758-4.

## Introduction

**Scheme 1:**
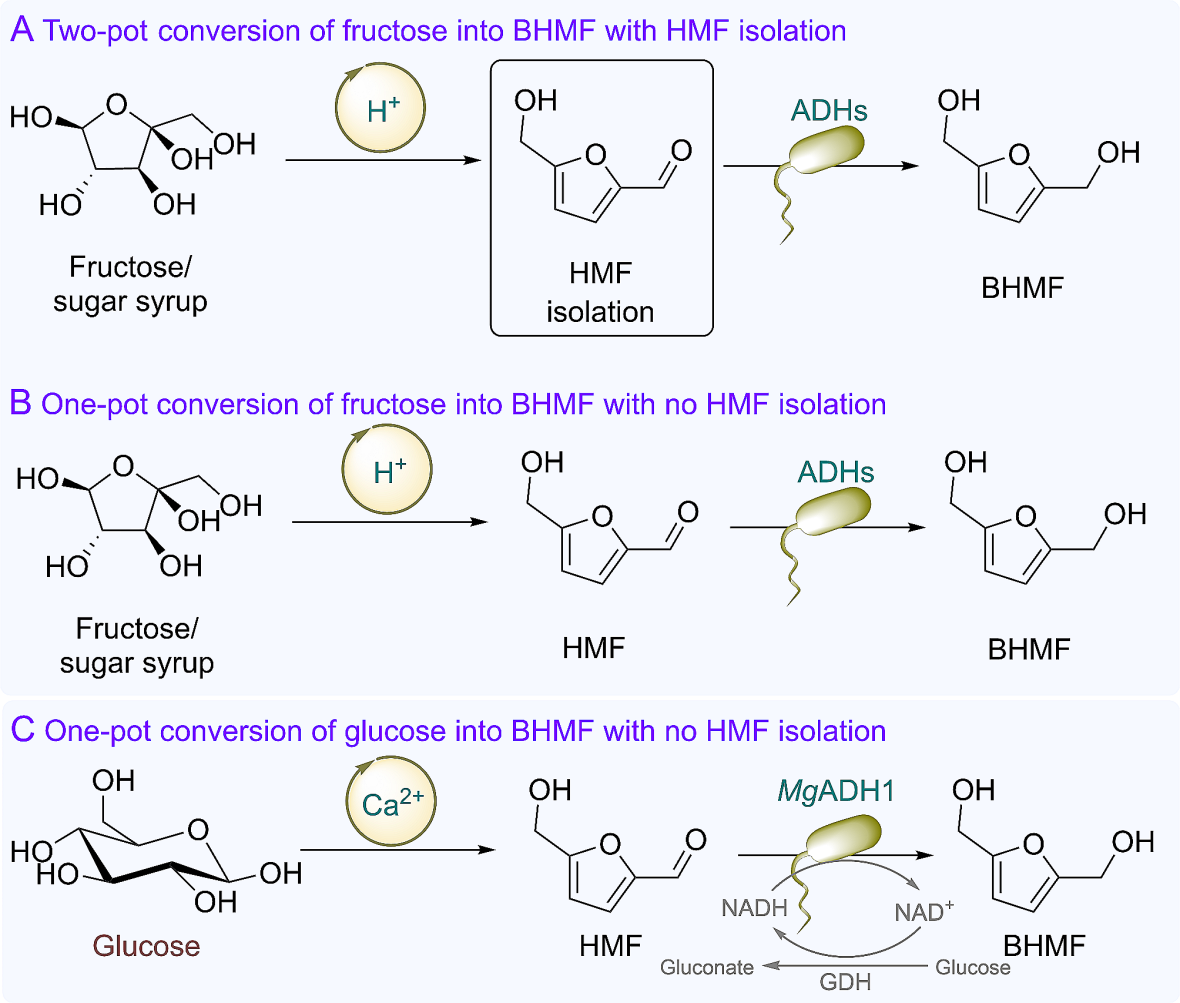
Catalytic conversion of biomass/HMF into BHMF. ADH, alcohol dehydrogenase; GDH, glucose dehydrogenase

Recently, concerns on fossil-based carbon resources risk and environmental issues (e.g., global warming) have been motivating chemists to focus on sustainable production of chemicals, fuels, and materials from renewable biomass, especially lignocellulosic materials (Figueirêdo et al. 2022; Sheldon 2018). 5-Hydroxymethylfurfural (HMF) that is obtained via dehydration of hexoses is an important platform biobased molecule linking commercially interesting chemicals with biomass. AVA Biochem (a subsidiary of the Swiss company AVA-CO_2_) has produced HMF with a capacity of 40 tons per annum since 2014, starting from unconcentrated sugar syrup (Klausli 2014). The global annual capacity of HMF is estimated to reach 100,000–1,000,000 tons if the chemical acts as a renewable commodity in the biobased chemical industry (Xu et al. 2020). The platform molecule can be converted into a number of valuable chemicals such as 2,5-furandicarboxylic acid (FDCA), 2,5-diformylfuran (DFF), and 2,5-bis(hydroxymethyl)furan (BHMF) via oxidoreduction reactions, due to its high chemical reactivity (Li and Zong 2022). From an economic perspective, sugar syrup and fructose remain too expensive to manufacture commercially interesting HMF-derived products if they serve as bulk chemicals. In contrast, glucose is much more attractive for producing HMF and derivatives owing to its lower cost, compared to fructose-containing materials. On the other hand, the vast majority of processes require pure HMF as substrate, mostly due to significantly deleterious effects of dehydration process-derived impurities such as residual catalysts/reactants/solvents and by-products on hydrogenation catalysts including chemical and biological ones (Fulignati et al. 2019; Hao et al. 2023; Turkin et al. 2022; Wu et al. 2023). As a result, HMF synthesis and its subsequent conversion were separately performed in most of previous studies on biomass valorization, requiring costly and time-consuming HMF purification (Scheme 1A) (Li and Zong 2022; van Putten et al. 2013). In contrast, few studies considered utilization of (semi)crude HMF for producing value-added furan-based products (Birmingham et al. 2021; Fulignati et al. 2019), or straightforward valorization of biomass (mostly fructose) via HMF as the intermediate (Scheme 1B) (Liu and Zhang 2016; Peng et al. 2022; Wrigstedt et al. 2017; Wu et al. 2023). In this work, HMF synthesis is integrated with its reduction in one pot, allowing for direct transformation of cheap glucose into value-added BHMF with no requirement of intensive HMF purification (Scheme 1C).

BHMF, a stable and symmetric furanic diol, is an important building block in the fields of biobased polymers (e.g., polyesters, polyurethanes and polyethers), biofuels and fuel additives, and fine chemicals (Hu et al. 2018; Post et al. 2023). The last decades have seen great advances in both chemo- and biocatalytic synthesis of BHMF (Du et al. 2023; Huang et al. 2022b; Li and Zong 2022; Zhao et al. 2023). In contrast to chemical routes, biocatalysis usually operates under mild conditions (ambient temperature and atmospheric pressure) and shows excellent selectivity, apart from catalysts being easily biodegradable and essentially nonhazardous and nontoxic. From a sustainability viewpoint, biocatalysis holds great promise in the biobased furan chemistry. One of the great hurdles for biocatalytic upgrading of biobased furans lies in the considerable toxicity and inhibition of furans towards enzymes/cells, particularly at high substrate concentrations (Ask et al. 2013; Cheng et al. 2020; Modig et al. 2002). In 2017, our group disclosed the first example of biocatalytic synthesis of BHMF from HMF using an HMF-tolerant yeast strain *Meyerozyma guilliermondii* SC1103 (Li et al. 2017). Then, many biocatalysts with good substrate tolerance and catalytic efficiency were discovered and developed for the reduction of biobased furans including HMF and furfural (Di et al. 2023; He et al. 2018; Pan et al. 2022; Xia et al. 2020). Most strikingly, a powerful *Escherichia coli* whole-cell catalyst harboring alcohol dehydrogenase (ADH) *Ec*YjgB and glucose dehydrogenase (GDH) was reported to enable efficient reduction of furans at the concentrations up to 1 M, providing approximately 15 g/L h BHMF productivity (Wu et al. 2023). The biocatalytic process can compete with chemically catalytic methods in terms of productivity. Herein, we present an HMF-resistant ADH from *M. guilliermondii* SC1103. Based on transcriptome analysis and real-time quantitative polymerase chain reaction (RT-qPCR), six candidate ADH genes were located from the genome of the strain. The six ADHs were expressed in *Saccharomyces cerevisiae*, followed by evaluation of catalytic performances in HMF reduction. An ADH (*Mg*ADH1) was identified, with an HMF-tolerant level of up to 400 mM.

Chemobiocatalysis incorporating chemo- and biocatalysts in a single vessel has recently emerged as a potent tool in synthetic chemistry (Bering et al. 2022; Gröger et al. 2023; Rudroff et al. 2018; Wang et al. 2024), due to the complementary advantages of the two types of catalysts. Besides, it may provide many profits such as higher overall yields, decreased solvent/time consumption and waste generation, and reduced work-up, due to no requirement of isolating intermediate(s). Although attractive, chemobiocatalytic transformations remain challenging, because of mutual inactivation and incompatibility issues between the two worlds of catalysts (Rudroff et al. 2018; Schmidt et al. 2018). Discovering/choosing appropriate catalysts represents a direct solution to such issues. Lin et al. reported that CaCl_2_-mediated glucose conversion into HMF in 2-methyltetrahydrofuran (MeTHF)/water biphasic system via sequential isomerization and dehydration, with >99% glucose conversion and 52% HMF yield (Lin et al. 2021). Ca^2+^ seems to be both environment- and enzyme-friendly. According to Antonucci et al. (Antonucci et al. 2011), MeTHF is considered negative for genotoxicity and mutagenicity. And the solvent has low water solubility (4 wt%, at 20 °C) and is easy to separate from aqueous phase; besides, it has moderate boiling point (80.2 °C), so it may be readily removed by evaporation (Bijoy et al. 2022). More importantly, MeTHF obtained from renewable sources (Gundekari and Karmee 2024; Huang et al. 2022a) is considered as a green solvent for organic synthesis (Pace et al. 2012). Considering the facts, we envisage that a straightforward and sustainable route for glucose conversion into BHMF may be implemented by combining CaCl_2_ and recombinant *S. cerevisiae* harboring *Mg*ADH1 (*S. cerevisiae*_*Mg*ADH1). Indeed, BHMF was obtained from glucose in this work with an overall yield of 42%.

## Results and discussion

### Identification of ADH for HMF reduction

Previously, we identified a robust ADH (*Mg*AAD1669) from *M. guilliermondii* SC1103, based on the functional annotations of the predicted coding sequences and random screening (Xia et al. 2020). To rationally and rapidly locate more potent ADHs responsible for HMF reduction, comparative transcriptome analysis of the yeast strain was performed with and without exposure to HMF (Fig. S1). It was found that 3251 genes showed differential transcriptome levels between the control and HMF groups, including 651 upregulated and 2600 downregulated genes. The upregulated genes mostly involved such biological functions as membrane transport, redox reactions, DNA repair and cold shock response. To verify comparative transcriptome results by RNA sequencing, six genes selected randomly were subject to RT-qPCR analysis (Fig. S2). Indeed, there is a close linear correlation between the results obtained by RNA sequencing and RT-qPCR, indicating the reliability of the transcriptome results by RNA sequencing. Therefore, six candidate genes encoding ADHs including *Mg*ADH1-4, short-chain dehydrogenase/reductase (*Mg*SDR) and xylitol dehydrogenase (*Mg*XDH) were identified (Table S2), based on their high transcriptome levels or significantly improved transcriptome levels upon exposure to HMF.

**Fig. 1:**
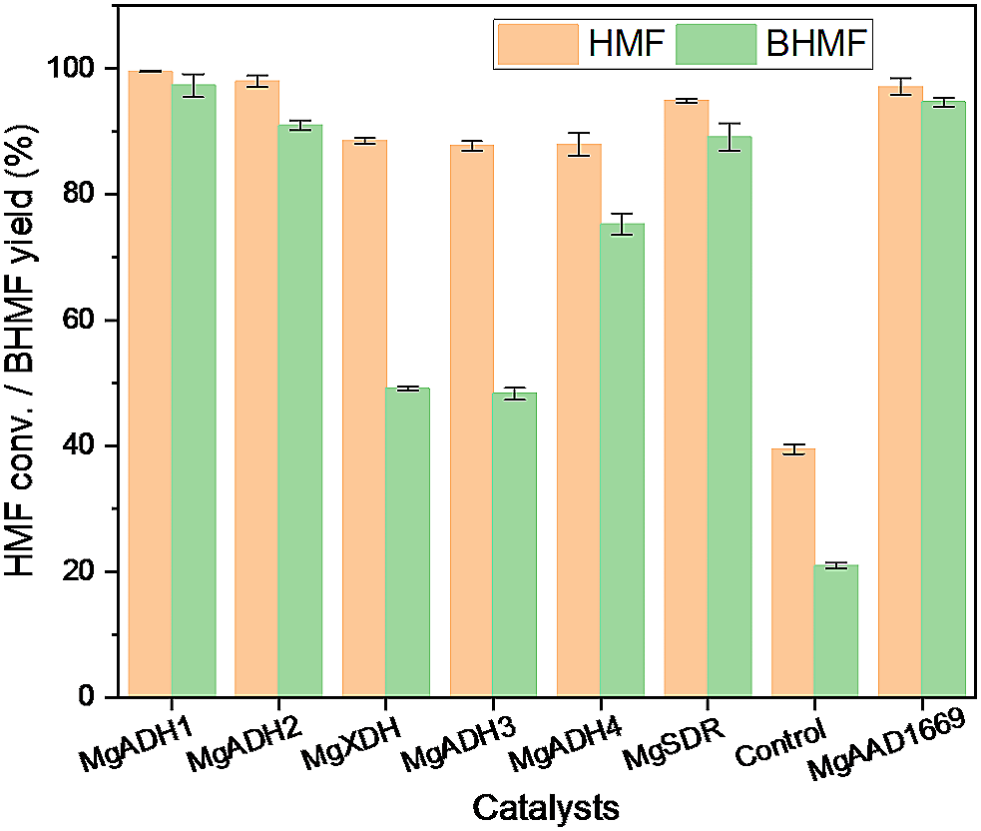
BHMF synthesis by *S. cerevisiae* harboring ADHs. Reaction conditions: 50 mM HMF, 30 mM glucose, 20 mg/mL cells, 4 mL phosphate buffer (0.1 M, pH 7), 30 °C, 200 rpm, 6 h

Then, the candidate genes were introduced into *S. cerevisiae*; the resulting recombinant yeasts were tested for HMF reduction, with comparison of *S. cerevisiae*_*Mg*AAD1669 (Fig. 1). The recombinant strain harboring empty plasmid acted as the control. Compared to the control, all recombinant yeasts provided higher HMF conversions as well as higher BHMF yields, indicating that these ADHs are active toward HMF. In addition, more than 90% BHMF yields were obtained in the cases of *Mg*ADH1, *Mg*ADH2 and *Mg*SDR. And even, the BHMF yield by newly identified *Mg*ADH1 was slightly higher than that by *Mg*AAD1669 (97% vs. 95%). To tap the potential of the newly identified enzyme for BHMF production, process optimization was subsequently conducted, together with codon optimization.

### Process optimization

**Fig. 2:**
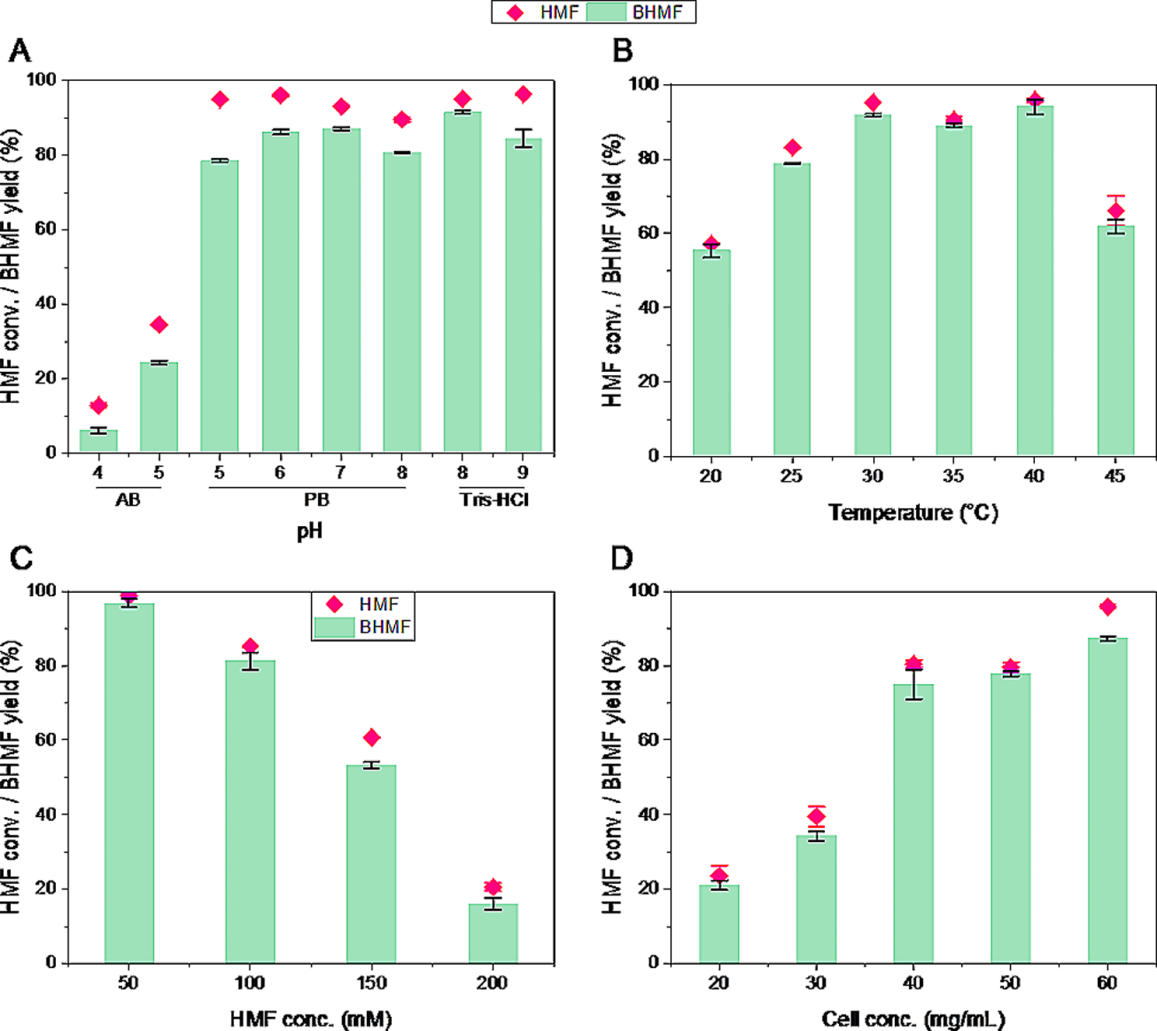
Process optimization: (**A**) pH; (**B**) temperature; (**C**) substrate concentrations; (**D**) cell concentrations. General reaction conditions unless otherwise stated: 30 mM HMF, 1 equiv. glucose, 20 mg/mL cells, 4 mL Tris-HCl buffer (0.1 M, pH 8), 30 °C, 200 rpm, 3 h; (**A**) 0.1 M acetate buffer (AB, pH 4 and 5), phosphate buffer (PB, pH 5–8), and Tris-HCl buffer (pH 8 and 9); (**B**) 20–45 °C; (**C**) 50–200 mM HMF, 12 h; (**D**) 200 mM HMF, 20–60 mg/mL cells, 12 h

Figure 2A shows the effect of pH on HMF reduction catalyzed by *S. cerevisiae*_*Mg*ADH1 cells. It was found that the cells displayed good catalytic performances within a wide pH range (pH 5–9), which is in good agreement with the previous results obtained by *S. cerevisiae*_*Mg*AAD1669 (Xia et al. 2020). More than 80% yields were obtained in most cases (Fig. 2A). Effect of reaction temperature on biocatalytic BHMF synthesis was studied (Fig. 2B). Good yields (89–94%) were achieved within 3 h at 30–40 °C. The higher temperature (45 °C) resulted in a reduced BHMF yield, likely due to thermal inactivation of enzymes. To identify optimal temperature for biocatalytic BHMF synthesis, the thermostability of *S. cerevisiae*_*Mg*ADH1 was evaluated (Fig. S3). As shown in Fig. S3, the cells retained good catalytic activity after incubation at 30 °C for 24 h, because up to 76% relative yield (compared to that by fresh cells) was observed when thermally treated cells were used for HMF reduction. Nonetheless, the incubation of the cells at 35 and 40 °C for the same time led to significant activity losses, which is clearly verified by low relative yields (less than 20%, Fig. S3). So, the temperature of 30 °C was considered optimal for BHMF synthesis.

Catalytic conversion of high concentrations of substrate is highly desired in the large-scale production, because it may not only lead to satisfactory productivity, but also offer high product titers and significantly simplify downstream processing process. So, the impact of the substrate concentrations on biocatalytic BHMF synthesis was investigated (Fig. 2C). As shown in Fig. 2C, the BHMF yield (97%) was good at the substrate concentration of 50 mM, whereas it sharply reduced to approximately 53% and even 16% at high concentrations of HMF (≥ 150 mM). It may be due to the great toxicity and inhibition of HMF toward cells (Cheng et al. 2020). Increasing catalyst concentrations is an effective strategy to enhance the reduction of HMF (Fig. 2D). For example, BHMF was obtained with 87% yield using 60 mg/mL cells at 200 mM HMF, which is much higher that using 20 mg/mL cells.

### Improving BHMF synthesis by codon optimization

**Fig. 3:**
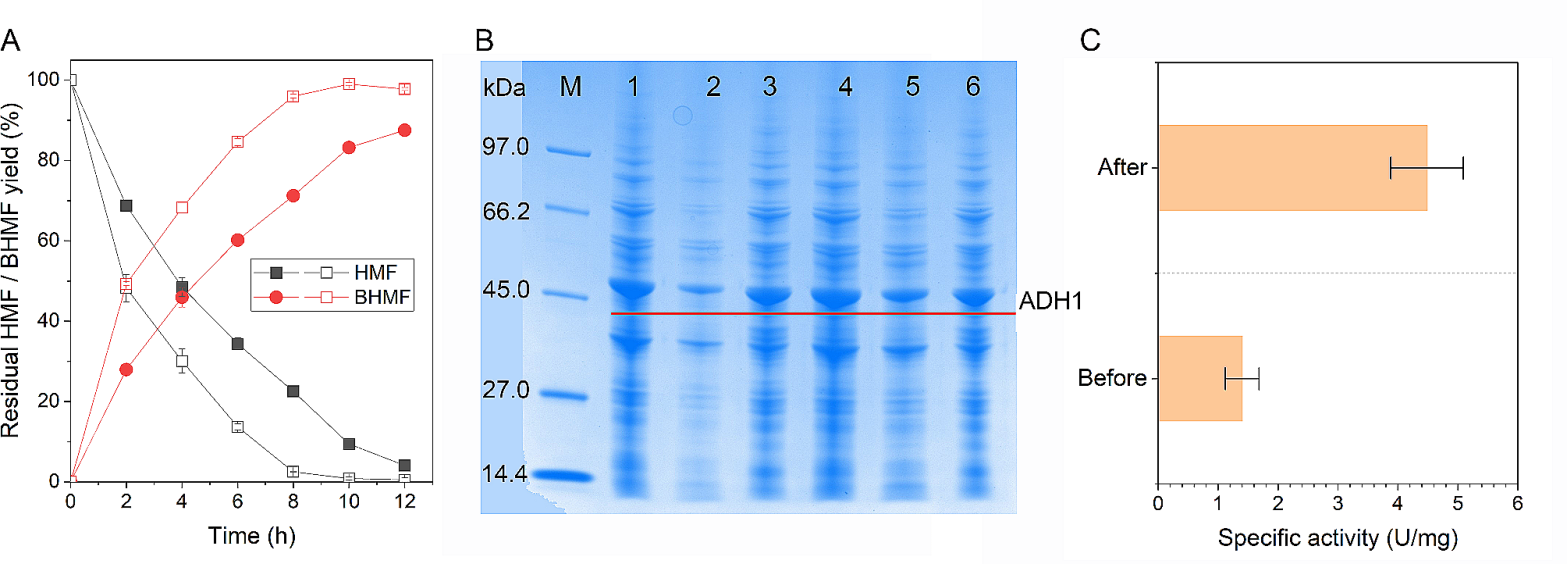
Comparison of BHMF synthesis before (solid symbols) and after (empty symbols) codon optimization of *Mg*ADH1 (**A**), SDS-PAGE analysis (**B**) and specific activity of the supernatant (**C**) before and after codon optimization. Reaction conditions (**A**): 200 mM HMF, 1 equiv. glucose, 60 mg/mL cells, 4 mL Tris-HCl buffer (0.1 M, pH 8), 30 °C, 200 rpm; (**B**) lane M: protein marker; before optimization: lane 1 (all proteins), lane 2 (supernatant), lane 3 (precipitant); after optimization: lane 4 (all proteins), lane 5 (supernatant), lane 6 (precipitant); (**C**) 5 mM HMF, 0.15 mM NADH, appropriate supernatant, Tris-HCl buffer (50 mM, pH 8), 35 °C

Although BHMF production was considerably improved upon process optimization (Fig. 2), soluble expression of *Mg*ADH1 in *S. cerevisiae* was not good (Fig. 3B). Codon optimization is a general strategy to improve heterologous expression of proteins (Fu et al. 2020; Lanza et al. 2014). To further enhance biocatalytic BHMF synthesis, therefore, this strategy was applied (Fig. 3). Figure 3A shows BHMF synthesis by recombinant yeast harboring codon-optimized *Mg*ADH1, with comparison of recombinant strain incorporating non-optimized enzyme. Strikingly, BHMF was produced with an almost quantitative yield within 10 h from 200 mM HMF by codon-optimized enzyme (Fig. 3A). In contrast, HMF reduction by non-optimized *Mg*ADH1 afforded BHMF with 88% yield within 12 h. Also, the BHMF productivity was considerably improved upon codon optimization (2.5 vs. 1.9 g/L h). To unveil the reason for enhanced BHMF production, SDS-PAGE analysis of proteins present in recombinant yeasts was conducted (Fig. 3B). As expected, codon optimization resulted in greatly improved soluble expression of the target enzyme (*Mg*ADH1). More importantly, the specific activity of the supernatant towards HMF was increased by 3-fold upon codon optimization, reaching 4.5 U/mg of protein (Fig. 3C). Thus, improved soluble expression of *Mg*ADH1 might make a contribution to enhanced BHMF synthesis.

### Optimized and fed-batch production of BHMF

**Fig. 4:**
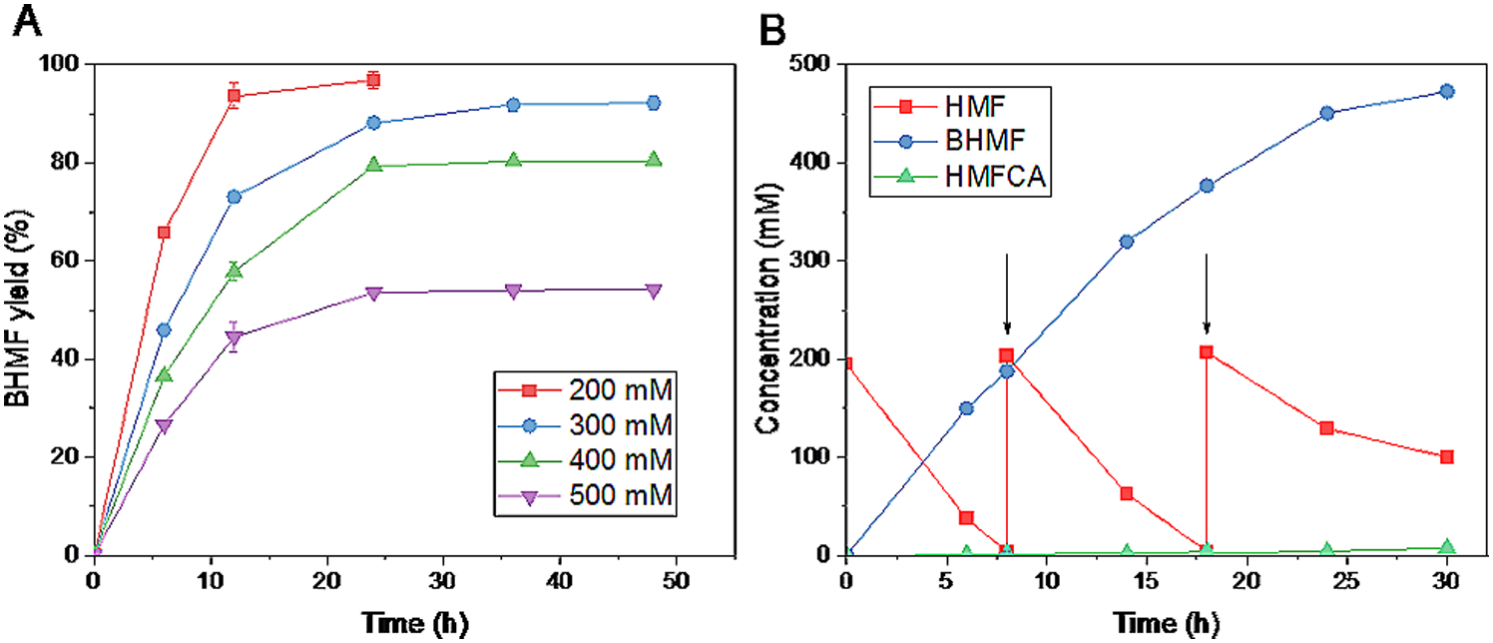
BHMF synthesis at high substrate concentrations (**A**) and fed-batch synthesis of BHMF (**B**). Reaction conditions (**A**): 200–500 mM HMF, 1 equiv. glucose, 60 mg/mL cells, 4 mL Tris-HCl buffer (0.1 M, pH 8), 30 °C, 200 rpm; (**B**) 8-mL scale, arrows refer to supplementation of approximately 200 mM fresh HMF and 1 equiv. glucose

Encouraged by the above results, the catalytic performances of *S. cerevisiae* cells harboring codon-optimized *Mg*ADH1 were re-evaluated at high substrate concentrations (Fig. 4A). As shown in Fig. 4A, BHMF was achieved with more than 90% yield within 36 h at the substrate concentration of 300 mM, along with the substrate conversion of 93% (Fig. S4). And even, 80% yield was obtained at 400 mM HMF. The HMF-tolerant level of *S. cerevisiae* _*Mg*ADH1 is higher than that of *S. cerevisiae* harboring *Mg*AAD1669, another ADH identified from the same strain previously by us (Xia et al. 2020). The former provided 80% BHMF yield at 400 mM HMF, while less than 20% yield was obtained by the latter under similar reaction conditions (Xia et al. 2020). In fact, this recombinant yeast strain is more resistant towards HMF than most whole-cell biocatalysts reported previously (Chang et al. 2021; He et al. 2018; Li and Zong 2022; Li et al. 2017; Xia et al. 2023; Xu et al. 2018), except for *E. coli*_*Ec*YjgB (Wu et al. 2023).

To produce high-titer product, batch-wise feeding of the substrate (approximately 200 mM) was performed (Fig. 4B). As shown in Fig. 4B, approximately 195 mM HMF was almost completely converted into BHMF within 8 h in the first batch. Also, the cells worked well in the second batch, and BHMF was obtained in quantitative yield within 10 h. Unfortunately, great inactivation of the cells was observed in the third batch, evidenced by significantly reduced HMF conversion (about 52%) within 12 h. Therefore, the long-term stability of the biocatalyst is unsatisfactory, and great effort may be donated to improve it in the future. In fact, it is also one of the long-standing challenges in biocatalytic conversion of toxic and inhibitory biobased furan platform chemicals (Li and Zong 2022). Overall, around 473 mM BHMF (60.5 g/L) was produced within 30 h, together with 7 mM 5-hydroxymethyl-2-furancarboxylic acid (HMFCA). The minor by-product formation might be attributed to the catalytic behavior of endogenous aldehyde dehydrogenase(s) in *S. cerevisiae* host cells, which may oxidize HMF using intracellular NAD(P)^+^. The overall BHMF productivity is around 2 g/L h. Notably, the productivity obtained in this work is lower than those by *Enterobacter ludwigii* YYP3 (Pan et al. 2022), E. *coli*_YjgB (Wu et al. 2023) and even the immobilized *M. guilliermondii* SC1103 (Xu et al. 2018). Therefore, further work is required for greatly improving the efficiency of the biocatalytic process by catalyst and process engineering in the future.

### Chemobiocatalytic BHMF synthesis from glucose

**Fig. 5:**
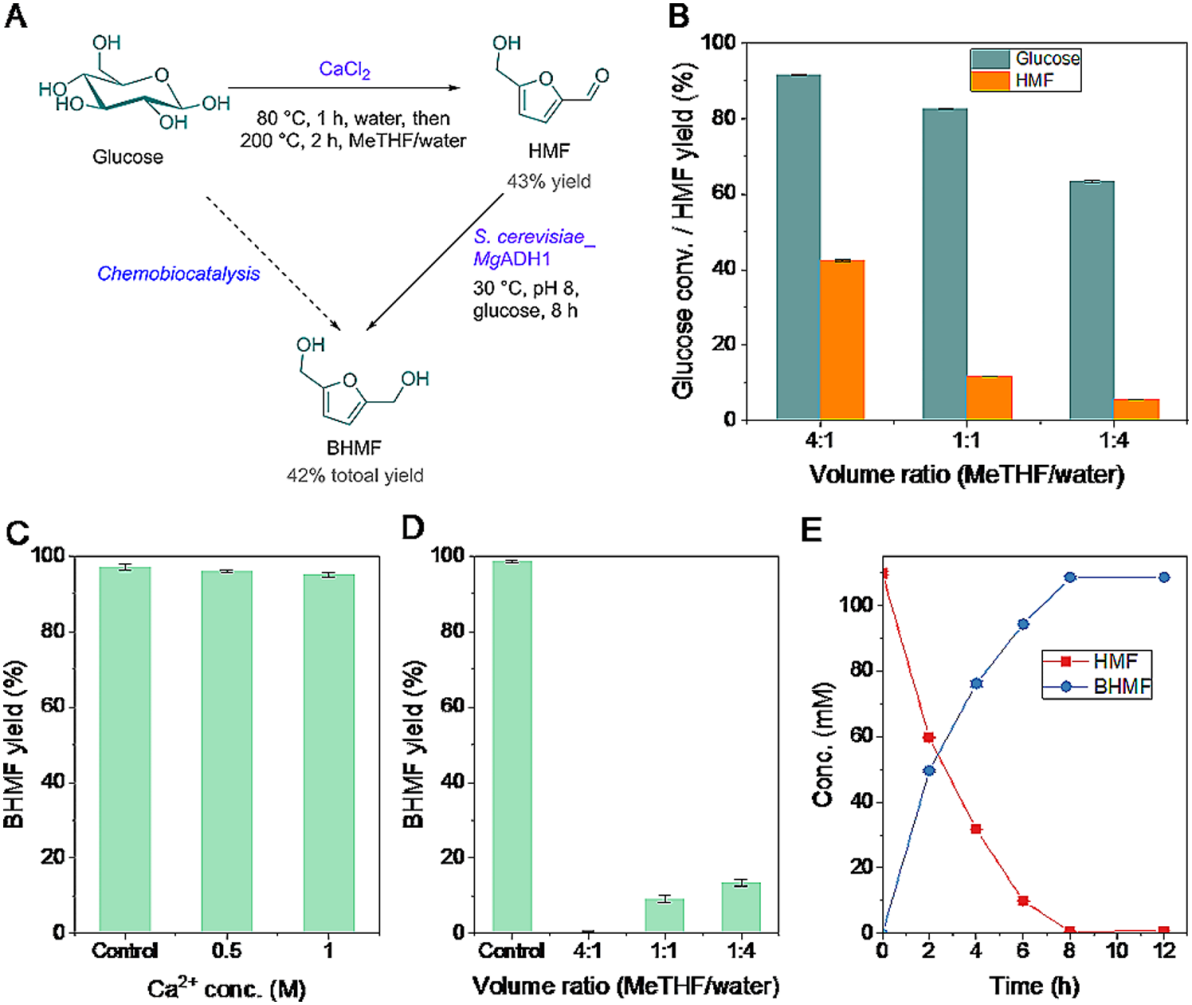
Twostep chemobiocatalytic synthesis of BHMF from glucose: (**A**) reaction scheme; (**B**) effect of MeTHF/water volume ratios on HMF synthesis; effect of Ca^2+^ concentrations (**C**) and MeTHF/water volume ratios (**D**) on biocatalytic BHMF synthesis; (**E**) biocatalytic production of BHMF from HMF obtained by chemocatalysis. Reaction conditions: (**B**) 0.25 g glucose, 0.6 g CaCl_2_, 2.5 mL deionized water, 80 °C, 1 h; then, adding 0.63-10 mL MeTHF, 200 °C, 2 h; (**C**) 50 mM HMF, 50 mM glucose, 0–1 M CaCl_2_, 60 mg/mL cells, 2.5 mL Tris-HCl buffer (0.1 M, pH 8), 30 ℃, 200 rpm, 6 h; (**D**) 50 mM HMF, 50 mM glucose, 60 mg/mL cells, 2.5 mL Tris-HCl buffer (0.1 M, pH 8), 0.63-10 mL MeTHF, 30 ℃, 200 rpm, 6 h; (**E**) approximately 110 mM crude HMF, 110 mM glucose, 60 mg/mL cells, 5 mL pH 8 aqueous solution, 30 ℃, 200 rpm

Finally, we attempted to produce BHMF from a cheap starting material glucose in a one-pot manner, by combining chemocatalytic isomerization/dehydration of glucose and biocatalytic reduction of HMF (Fig. 5). According to Lin et al. (Lin et al. 2021), two-stage conversion of glucose into HMF was performed by CaCl_2_ (Fig. 5A), namely aqueous isomerization of glucose at 80 °C for 1 h, followed by dehydration of fructose in MeTHF/water biphasic system at 200 °C for 2 h. In the biphasic system, MeTHF acted as a HMF reservoir, since it was capable of extracting HMF from aqueous phase, reducing side reactions associated with HMF and thus improving HMF yield and selectivity. The effect of MeTHF/water volume ratios on HMF production was examined (Fig. 5B). It was found that the volume ratios exerted a remarkable influence on HMF production as well as glucose conversion. A satisfactory HMF yield (approximately 43%) was obtained in MeTHF/water (4:1), while the exploitation of MeTHF/water (1:4) resulted in 6% HMF yield, along with 63% glucose conversion. Therefore, HMF yield and selectivity may be improved by increasing the volume ratios of organic/aqueous phase.

Chemobiocatalytic transformation is generally performed in a single reaction vessel, without isolation of intermediate(s) (Hao et al. 2023; He et al. 2017). The components such as remaining catalyst(s), solvent(s), and reagents in the preceding catalytic system may have effects on the activity of the sequential catalyst. So, the effects of Ca^2+^ concentrations and MeTHF/water volume ratios on biocatalytic BHMF synthesis were studied prior to integrated catalysis (Fig. 5C and D). As shown in Fig. 5C, S. *cerevisiae*_*Mg*ADH1 cells worked well in the presence of up to 1 M Ca^2+^ in HMF reduction, giving 95% BHMF yield. So, Ca^2+^ is benign to the recombinant yeast. Unfortunately, low BHMF yields (< 20%) were observed, regardless of MeTHF/water volume ratios (Fig. 5D). It suggests that the strain is highly sensitive to MeTHF inactivation, although many biocatalytic transformations were successfully implemented in this organic solvent (Gao et al. 2013; Hoyos et al. 2011; Hu et al. 2016; Shanmuganathan et al. 2010). Therefore, MeTHF removal is required prior to biocatalytic reduction. Upon HMF synthesis and then MeTHF evaporation, the mixtures were diluted one-fold by pH 8 Tris-HCl buffer, followed by pH tuning to pH 8. The cells and glucose were supplemented to initiate biocatalytic HMF reduction at the final substrate concentration of approximately 110 mM (Fig. 5E). Pleasingly, HMF was almost quantitatively converted into BHMF within 8 h. BHMF was obtained with a total yield of approximately 42%, starting from glucose.

## Conclusion

In summary, we identified a powerful ADH (*Mg*ADH1) from *M. guilliermondii* SC1103 for HMF reduction. The HMF tolerant level of recombinant *S. cerevisiae*_*Mg*ADH1 was significantly improved to 400 mM upon codon optimization of the exogenous gene, due to enhanced soluble expression of the enzyme. A high titer of BHMF (60.5 g/L) was produced in the fed-batch process. Unfortunately, the long-term stability of the whole-cell catalyst is unsatisfactory, and great effort may be made to address this issue by cell immobilization as well as removal of reactive oxygen species caused by furans in the future. A sustainable route was constructed for direct conversion of cheap glucose into BHMF by combining CaCl_2_ and *S. cerevisiae*_*Mg*ADH1, without tied and time-consuming purification of the intermediate HMF. BHMF was obtained with a good overall yield (42%). More importantly, the chemobiocatalytic process is advantageous over the previous ones with regard to process economics, because glucose is much cheaper than fructose/sugar syrup and HMF. The present work may lay the foundation for sustainable production of BHMF and derivatives on large scale.

## Materials and methods

### Materials

*M. guilliermondii* SC1103 isolated previously by our group (Li et al. 2017) was maintained in our lab. *S. cerevisiae* INVSc1 and the plasmid pYES2 was purchased from Vazyme Biotech Co., Ltd. (Nanjing, China). HMF (98%), BHMF (98%), NADH (98%), and MeTHF (99.5%) were purchased from Aladdin Biochemical Co., Ltd. (Shanghai, China). PrimeSTAR DNA polymerase was purchased from TaKaRa Biotechnology Co., Ltd. (Dalian, China). Seamless cloning kit, galactose (98%), raffinose (99%), DNA marker and protein marker were purchased from Sangon Biotech (Shanghai, China).

### Total RNA extraction, RNA sequencing and RT-qPCR

*M. guilliermondii* SC1103 cells were cultured in 30 mL YPD medium (1% yeast extract, 2% peptone, and 2% glucose) at 30 °C and 200 rpm for around 16 h. Then, 100 mL YPD medium was inoculated with 1 mL of the above culture in the presence or absence of 50 mM HMF. Upon cultivation at 30 °C and 200 rpm for 4 h, cells were harvested by centrifugation (8000 rpm, 5 min, 4 °C) and washed twice with deionized water. Total RNA extraction from the cells was performed by Sangon Biotech (Shanghai, China), according to a previous method with Trizol (Sultan et al. 2014). Also, RNA sequencing and RT-qPCR were conducted by Sangon Biotech (Shanghai, China).

### Cloning and heterologous expression of ADHs in *S. Cerevisiae*

The genomic DNA was extracted as the template from *M. guilliermondii* SC1103 using rapid yeast genomic DNA isolation kit (Sangon Biotech, Shanghai, China). Primers were designed on the basis of the gene sequences of ADHs (Table S1) and synthesized by Sangon Biotech (Shanghai, China). The ADH fragments were amplified with the abovementioned template and primers. Linearized plasmid was generated using inverse-PCR on the basis of pYES2 sequence (Table S1). The ADH fragments were cloned to the plasmid pYES2 by ready-to-use seamless cloning kit (Sangon Biotech, China). The resulting recombinant plasmids were transformed into *S. cerevisiae* INVSc1, followed by pre-cultivation in 30 mL of the complete synthetic medium without uracil (SC-U) supplemented with 2% glucose at 30 ℃ and 200 rpm overnight. The cells were isolated by centrifugation, followed by resuspension in 1 mL of induction medium (SC-U medium supplemented with 2% galactose and 1% raffinose). Then, the cells suspended were inoculated into 100 mL of induction medium with a final OD600 value of 0.4, and the cultivation was performed at 30 ℃ and 200 rpm for 36 h. The cells were harvested by centrifugation (8000 rpm, 4 min, 4 ℃) and washed twice with deionized water.

### Biocatalytic reduction of HMF

Typically, 20 mg/mL of cells (wet weight) were added into 4 mL Tris-HCl buffer (0.1 M, pH 8) containing 30 mM HMF and 1 equiv. glucose, followed by incubation at 30 °C and 200 rpm. Aliquots were withdrawn from the reaction mixtures at specified time intervals and diluted with the corresponding mobile phase prior to HPLC analysis. The conversion was defined as the ratio of consumed amount of HMF to its initial amount (in mol). The yield was defined as the ratio of the measured product amount to the theoretical product amount based on the initial amount of HMF (in mol). All experiments were conducted in duplicate.

### Chemobiocatalytic conversion of glucose into BHMF

The synthesis of HMF from glucose was performed according to a previous method (Lin et al. 2021), with some modifications. To 2.5 mL of deionized water were added 0.25 g glucose and 0.6 g CaCl_2_, followed by incubation with stirring at 80 °C for 1 h. Then, 0.63-10 mL of MeTHF was supplemented to the mixtures, followed by reaction with stirring at 200 °C for 2 h. Upon MeTHF evaporation under the vacuum, the reaction mixtures were diluted one-fold by Tris-HCl buffer (0.2 M, pH 8), and pH was tuned to 8. The cells and glucose were supplemented, and the final volume was approximately 5 mL. The reaction mixtures containing 60 mg/mL cells, 110 mM HMF and 110 mM glucose were incubated at 30 ℃ and 200 rpm. The yield and conversion were determined by HPLC, based on the corresponding calibration curves.

### Enzyme assay

The ADH activity was assayed according to a recent method (Wu et al. 2023), with minor modifications. Briefly, crude enzyme solution was added into 1.5 mL Tris-HCl buffer (50 mM, pH 8) containing HMF (5 mM, final concentration) and NAD(P)H (0.15 mM, final concentration), and the decrease in absorbance at 340 nm (ε of NAD(P)H, 6220 M^− 1^ cm^− 1^) was determined in a UV2550 UV-visible spectrophotometer (Shimadzu, Japan) at 35 ℃. One unit was defined as the amount of enzyme that oxidizes 1 µmol NAD(P)H per minute under the assay conditions.

### Analytic methods

The reducing sugar contents were determined by the dinitrosalicylic acid (DNS) method (Ghose 1987; Ren et al. 2016). Quantification of HMF and BHMF was performed on a Zorbax Eclipse Plus C18 column (4.6 mm × 250 mm, 5 μm, Agilent, USA) with Waters 996 photodiode array detector (Waters, USA). The mobile phase is the mixture of acetonitrile and 0.4% (NH_4_)_2_SO_4_ aqueous solution of pH 3.5 (1/9, v/v) at a flow rate of 0.6 mL/min. The retention times of BHMF (224 nm) and HMF (284 nm) are approximately 8.5 and 10.3 min, respectively.

### Electronic supplementary material

Below is the link to the electronic supplementary material.

Supplementary Material 1

Supplementary Material 2

## Data Availability

All data generated or analyzed during this study are included in this published article (and its supplementary information files).
