# Supplementary material for: A straightforward chemobiocatalytic route for one-pot valorization of glucose into 2,5-bis(hydroxymethyl)furan
Source: Bioresour Bioprocess. 2024 Apr 18;11(1):38. doi: 10.1186/s40643-024-00758-4 (PMC11026326; doi:10.1186/s40643-024-00758-4)
Supplement: Supplementary file 1 — Supplementary Material 1 [file 40643_2024_758_MOESM1_ESM.pdf]

Supplementary Information

A straightforward chemobiocatalytic route for one-pot valorization of glucose into 2,5-bis(hydroxymethyl)furan

Xuan-Ping Liao, Qian Wu, Min-Hua Zong, Ning Li*

School of Food Science and Engineering, South China University of Technology, 381 Wushan Road, Guangzhou 510640, China
*Corresponding author, Email: lining@scut.edu.cn

Table S1 Primers used in this work

Primers' name	The sequence of primers (5'→3')
ADH1 F	GTACCGAGCTCGGATGAAAGCAGCAGTA
ADH1 R	CCCTCTAGATGCATTTAGTTGGCTCTGAAG
ADH2 F	GGTACCGAGCTCGGATGAGAGCAATTG
ADH2 R	CCTCTAGATGCATTTAATTAGCATAACGTGGTG
ADH3 F	GTACCGAGCTCGGATGTCCGAAATAAACT
ADH3 R	CTCTAGATGCATTTATGCCTTGGCCCCA
ADH4 F	GGTACCGAGCTCGGATGACAGTCAAAGAA
ADH4 R	CCTCTAGATGCATTTATTCCGGATCAGCAAG
SDR F	GTACCGAGCTCGGATGGCAAAACAAGT
SDR R	CCTCTAGATGCATCTACCACGGAACCT
XDR F	TACCGAGCTCGGATGACTCCCAAC
XDR R	CCTCTAGATGCATTTACTCGGGACCATCT
Linearized vector F	CCGAGCTCGGTACCAAGCTTAATATTC
Linearized vector R	ATGCATCTAGAGGGCCGCATCATGTAA

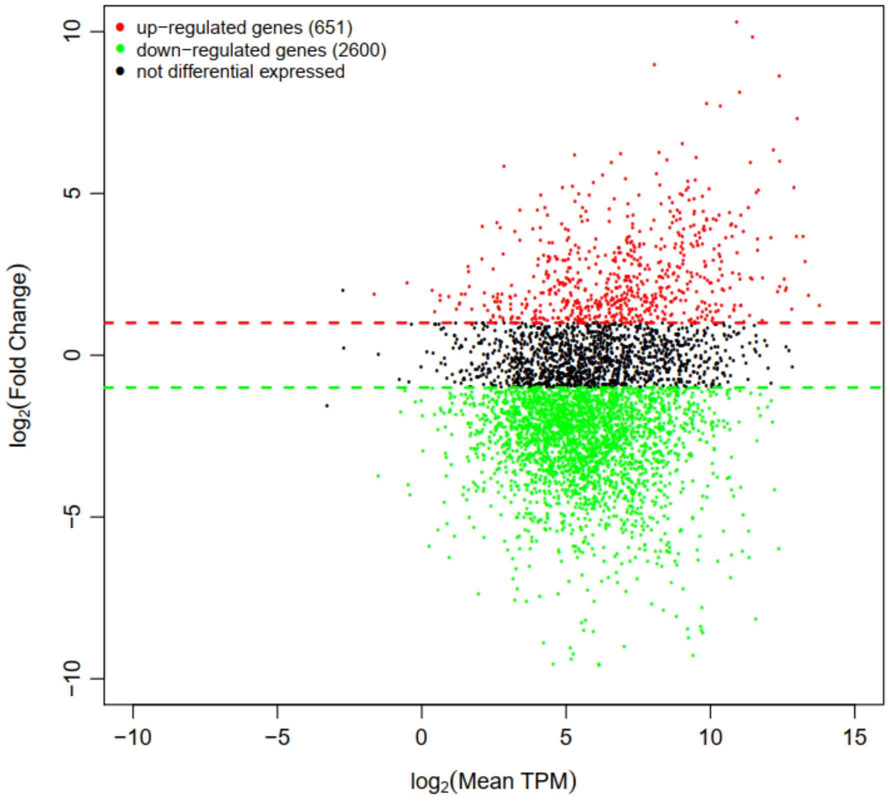

Fig. S1 Comparative transcriptome analysis of *M. guilliermondii* SC1103 under and without HMF stress.

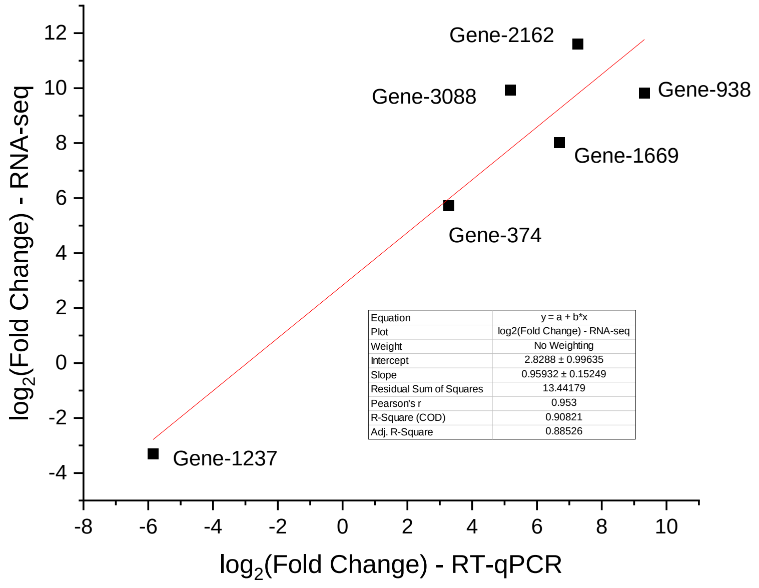

Fig. S2 Correlation of differential transcriptome levels obtained by RNA sequencing and RT-qPCR.

Table S2 Identification of candidate genes

Candidate gene	Relative transcript level		log_2(Fold Change)
	Without HMF stress	Under HMF stress	
*Mg*ADH1	2234.98	1968.4	-0.18
*Mg*ADH2	550.11	483.94	-0.18
*Mg*XDH	341.1	931.73	1.45
*Mg*ADH3	1.71	90.92	5.73
*Mg*ADH4	0.43	420	9.93
*Mg*SDR	0.19	609	11.64

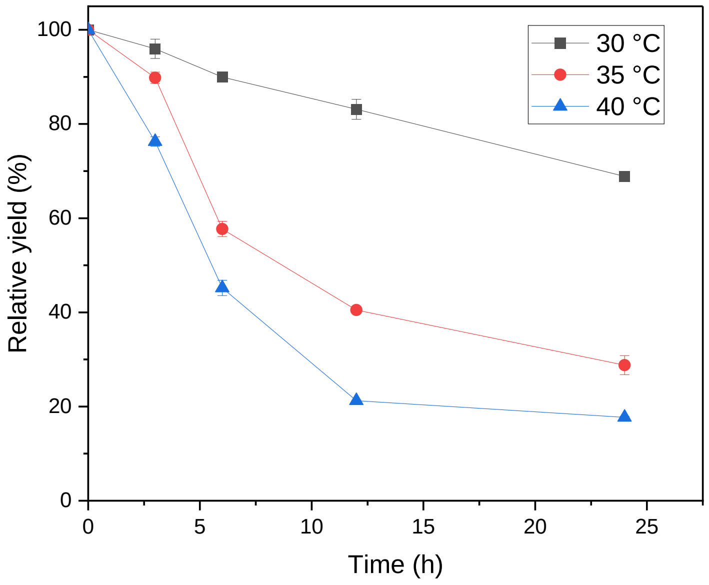

Fig. S3 Thermostability of *S. cerevisiae_Mg*ADH1. After the cells (20 mg/mL) were incubation in 4 mL Tris-HCl buffer (0.1 M, pH 8) at designated temperature for designated period, 30 mM HMF and 30 mM glucose were supplemented to initiate the reaction at 30 °C and 200 rpm. The yields were determined after 3 h. The yield obtained by fresh cells worked as the control.

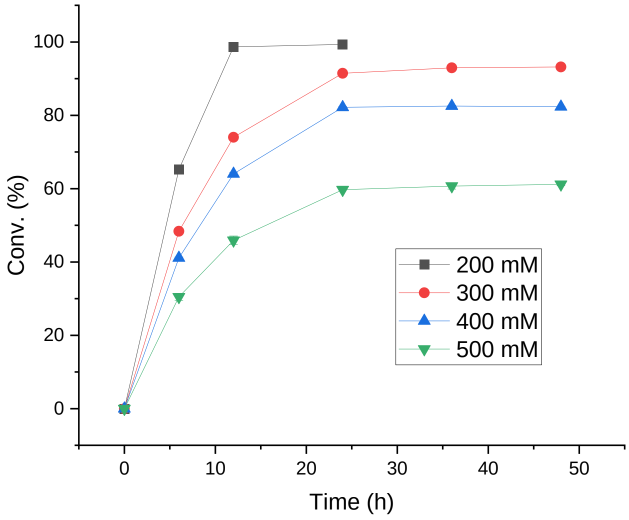

Fig. S4 BHMF synthesis at high substrate concentrations. Reaction conditions are the same as those in Fig. 4A.

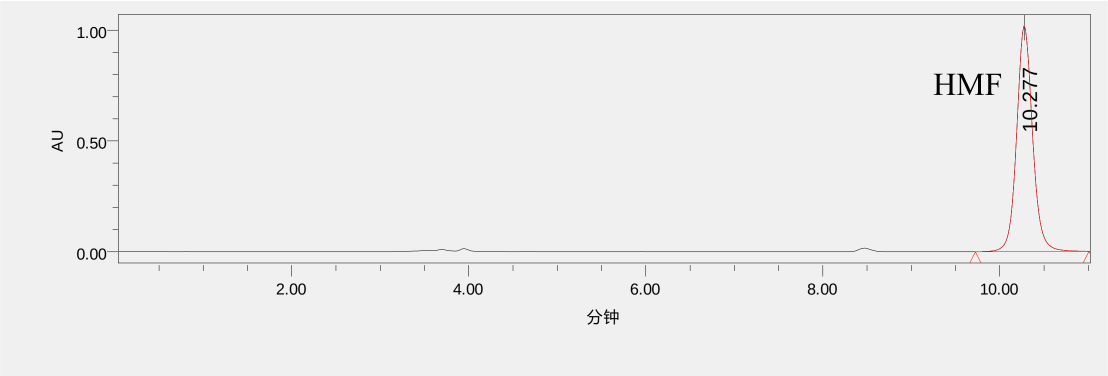

Fig. S5 HPLC chromatogram of the reaction mixture in HMF synthesis from glucose

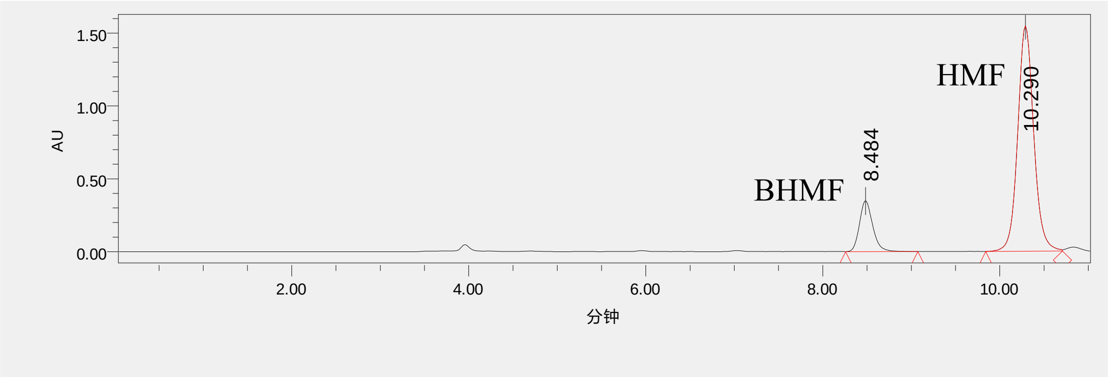

Fig. S6 HPLC chromatogram of the reaction mixture in HMF reduction

Full-length gene sequences of ADHs

ADH1 (before codon optimization)

ATGAAAGCAGCAGTACTTACTGGAAACCCAGAAAACAACTGGACGGAAATCAAGGAC
CTTCCCAAACCAACTATTAGAGACGATCAAATCTTAATCAAGCCAATTGCTTATGCTGC
AAATCCAACCGACTGGAAACACAATTCGCCCGAATTTGCTGGTAATGTTTCTGGTTCTG
ATGCCAGTGGATATGTTGAAGAAGTAGGTTCCAAAGTCTCTGGAATCCAAGTGGGTGA
CATTGTTTCTGTCACTATGCACGGAGGTTTCAACAAGGACAATTCGGCGTTTGCAGAGT
ATGTTGCTGCCGTTCCTGGATTCACCATCAAATACGACAAAAATATTGTGAAGGATGCA
GAATTGAAGGTGGGCGATTACGAAGGTAGCAAAATCACCAATTTCGAACAGGCTGCTT
CCGTCACTTTGGGTTTGTCAACGGTGGTTTTGTCGTTTGCTGGTTTGATGAACATTAAC
AAAGATGTTCTGGCCAACAAAGGTAAGTCTATTCTTATTTGGGGAGGTGCCACTGCCA
CGGGTGTTTTGGCAATTCAAATTGCCAAGTTGGCATTTGGTCTCAAGGTCATTACCACT
GCTTCAAAGAAGAACCATGAGTACTTGAAGAGCCTTGGAGCAGATGCTGTTTTTGACT
ACAACGATTCGAATGTGGTCGAAGCTATCAAGAGTGAGGCTGGCGACTCCATTGCTTA
TGCTTTGGACACCGTTGCCAACACTGACACTTTCCAAAAAACTTATGATGCAACCGCC
AATTCAAAGCATGTTGAACTCGACAACTTGCTTATGCTCGACAAGAGCCAAATCAAGA
CAGATTCTTCCAGATCGGTATCTTACCACAAGACACTCATGTACGGTGCCACAGGTGAT
GCCTTTAGCATTTGGGGTATGGACTTCCCTACCAACCATGTTTCTACCAAGAGATATCTT
GACTACTGGTTGAACGAACTTCCTAAATACATTCCTCATATCAAGTCTGCAAACTTGAA
GGTCATCAAGCCTGGATTGGAAAGCGTTAACGAAGCTTTGCAGTTGTTGCACGATAAC
AAGGTAAGCGGCCAGAAGATTGTCTTCAGAGCCAACTAA

ADH1 (after codon optimization)

ATGAAGGCTGCTGTTTTGACTGGTAACCCAGAAAACAACTGGACTGAAATCAAGGAC
TTGCCAAAGCCAACTATCAGAGACGACCAAATCTTGATCAAGCCAATCGCTTACGCT
GCTAACCCAACTGACTGGAAGCACAACTCTCCAGAATTCGCTGGTAACGTTTCTGGTT
CTGACGCTTCTGGTTACGTTGAAGAAGTTGGTTCTAAGGTTTCTGGTATCCAAGTTGG
TGACATCGTTTCTGTTACTATGCACGGTGGTTTCAACAAGGACAACTCTGCTTTCGCT
GAATACGTTGCTGCTGTTCCAGGTTTCACTATCAAGTACGACAAGAACATCGTTAAGG
ACGCTGAATTGAAGGTTGGTGACTACGAAGGTTCTAAGATCACTAACTTCGAACAAG
CTGCTTCTGTTACTTTGGGTTTGTCTACTGTTGTTTTGTCTTTCGCTGGTTTGATGAACA
TCAACAAGGACGTTTTGGCTAACAAGGGTAAGTCTATCTTGATCTGGGGTGGTGCTAC
TGCTACTGGTGTTTTGGCTATCCAAATCGCTAAGTTGGCTTTCGGTTTGAAGGTTATCA
CTACTGCTTCTAAGAAGAACCACGAATACTTGAAGTCTTTGGGTGCTGACGCTGTTTT
CGACTACAACGACTCTAACGTTGTTGAAGCTATCAAGTCTGAAGCTGGTGACTCTATC
GCTTACGCTTTGGACACTGTTGCTAACACTGACACTTTCCAAAAGACTTACGACGCTA
CTGCTAACTCTAAGCACGTTGAATTGGACAACTTGTTGATGTTGGACAAGTCTCAAAT
CAAGACTGACTCTTCTAGATCTGTTTCTTACCACAAGACTTTGATGTACGGTGCTACT
GGTGACGCTTTCTCTATCTGGGGTATGGACTTCCCAACTAACCACGTTTCTACTAAGA

GATACTTGGACTACTGGTTGAACGAATTGCCAAAGTACATCCCACACATCAAGTCTGC
TAACTTGAAGGTTATCAAGCCAGGTTTGGAATCTGTTAACGAAGCTTTGCAATTGTTG
CACGACAACAAGGTTTCTGGTCAAAAGATCGTTTTCAGAGCTAACTAA

ADH2

ATGAGAGCAATTGTTCTTCATGGTGTTAAAGATTTAAAATTTCACAGCGATTATCCGG
AGCCGCAGCTACAATCTCCAACAGATGTCAAGATAAAGGTTGATTATTGCGGTATCT
GCGGCTCTGATTTGCACGAATATTTGGATGGGCCAATTTTCTTTAAAAACAAACGAAA
CGAAATCTCCAATAAGGAAAACATACAATGTATGGGTCATGAAATGTGTGGAGAAAT
TGTGGAATTGGGCGCCAAGGTGAACCCAGAATACAAAGTTGGCCAGAAAGTGGTGGT
GGCGCCAACCGGAACTTGCTTGGATCGACCTCGGTTTCCTGATGCACCCAACGCAAA
GAAGCAGCCCTGCAATGCATGTAGTGAAGGATCATATAATGCGTGCGACTATATTGC
GTTTACCGGATTGGGATTCGAAGACGGCGGATTTGGCGACTACTGCGTTGTGGGCGA
CAACCACATTGTGCCGTATCCACCATCGGTGATTCCGGTGGACGTGGCGGCATTGATT
GAGCCGCTTGCAGTTGCATGGCATGCAGTGCGCATCGCTAAGCTAAATGAGGGCGAA
CTGGCGTTGGTGCTTGGCGCGGGACCCATTGGTCTCGTGATGATTCTTGCGTTAAAAG
CACACAAGGCCGGAAATATTGTCGTGAGTGAGCCGGCAGAAGCTCGCAGAAAGTTGG
CAGAGTCTTTTGGCGTGCAGACTTTTGACCCCACAAATTACGAATCGGTAGACCAGTC
GGTTACCGCGCTTAAAAAGTTGACCAATGACGGATTTGGCTTCCACCATTCTTTCGAC
TGTTCCGGCATTCCGGTCTCGTTCGAGGTTTCTTTGAAGGCATTACGAACCACCGGAG
TAGCCACCAACGTTGCCATTTGGCCGGAAAAACCGGTGGATTATTTTCCCATGGAAAT
CACATTGCATGAAAGAACCATCAATGGATCCATGTGCCATACTCGTGAAGACTTTGTA
AACGTTGTCAAGGCATTCGAAGCCAAACTTGTAGACATTGACCAGGTGAGAACTCTA
ATAACCGGAATTGTGTCGGTTGAAGACGGAATAGAAAAGGGCTTTAACGAGCTTATC
AACCACAAGGAGAAACACATCAAGATACTTGTATCACCACGTTATGCTAATTAA

ADH3

ATGTCCGAAATAAACTTCACTCCACTTAAAAACACCAAGGTTTTCCAGCCGATTCAAG
TTGGAAAAAACCTCCTTTCCAACCGAATTTTTATGCCCCCTCCACAAGAACCAGAGC
ATTGGACGATCGGACTCCTTCGAACTTGCAATTGCGCAACTACGACGAAAGAACAAA
GTATGCTGGTTCGCTTGTGGTCACCGAAGCCACTTTTTCATTTCCTCAGGCCGGAACC
ATGGCAGGAGTTCCGGGAATATATACCCCAGAACACACCAAAGGATGGAAAAAAAT
TGTGGATAAGGTTCACGAGAACAATTCGTTTATTGCAATTCAGCTTTGGAACTTGGGT
CGACTTGATAACCCCAAAGATTTGAAGGCAGTAGGCTTACCCTACTTGGCACCTTCAG
CCATTTATCCCGACAAAGATGCTCGGGAAGAAGCCGAGGCTGCGAATAATCCTATTA
GAGCATTGACCGAAGAGGAAATCCACAACCAGATTTATGTGGAATACACCACGGCTG
CAAAAAATGCCGTTGAGGCTGGGTTCGACTACTTGGAGTTACATGGTGCTCACGGCT
ACTTGTTGCACCAATTCTTGGAAGATACCTCCAACCAAAGGACAGACAAGTATGGTG

GATCGGTAGAGAACAGAGCCAGGTTTGTGTTGGAGCTCATTGACCATCTTATTCCGAT
AGTTGGTGCTGACAAACTTGCTATTCGTCTTTCTCCATGGGTGACTATTAAAGGTATG
CCTGGTATTCATGGTGATACCCATCCATTGACCACCTACAGCTACTTGCTACACGAGC
TTGAAAAACGGGCTAAAGCCGGGAATCGGTTAGCCTACATTTCCATTGTAGAGCCTA
GAGTCAATGGGTCTACTACTCTTGAAACCAAAGACCAAACTGGAGACAATGGTTTTG
TTGAAGATATTTGGAAGGGAACCATTCTCAAGGCGGGAAACTACACATATGATGCTC
CAAAGTTCAATCTGGTGATTAAAGACGTTGAAAACGACCGTACGTTAGTCGGGTTCA
GTCGCTATTACGTCTCGAATCCAGACTTGGTTCAGCGATTGAAGGACGGGACTCCACT
CAAGCCTTACGACCGTTCTCTCTTTTATCGAAAGGACGACTGGGGATACAATACTTAT
CCATACGAGGGACAAACAGAAGAGGAAATAGAGGCTGCAAAAAACAGAAAGCCAA
AGCCTATTGGGGCCAAGGCATAA

ADH4

ATGACAGTCAAAGAATCTAGAGTATTTTACCTCAATCAGCAAACTGAAGGACTTCCC
AATGTTGAATTGGGTGAGCCTAACTCCACTTTTAGGCTCGTAACAGAAGAGTTGCAGC
CATTAAAAGAAAATGAAGTATTGGTGAAATCTCTCTATTTTTCCAACGATCCGCTCCA
ATTGGGCTGGATCAGAAAGGCAAGCTACGAAAACGCTGGTATTAGGCGGATTTTTCC
GGGTGAGCCTATGGGTGGGTTCGGTTTGGGGCAGGTTATTGAGTCAACGGCAGACCA
ATATAAGCCTGGGGACATTGTTAATGGTGCTTTGAACTGGGCAGATTATAGTATTGTA
AAGGCCGGAGCTCTTACAGGCAAAATTCCAGATTCTTCATTGCCTTTGACTACCTCGT
TATCGACTATAGGACTCACCGGTTTGACTGGCTACTTTGGAATATACGGCCAGGTCAA
AGAGGGAGATACCGTAATTGTATCGGCAGCTTCTGGGGCCACGGGTTTGGTAGCGGT
TCAAGTTGCTAAGGCTTTAGGATGTCGGGTTGTTGGGATCACTGGAAGCGACGAAAA
GTGCAAATTTGTTGTAGGACTTGGAGCAGATGCGTGTGTCAATTACAACGACCCTGA
GTTGATTGAAAAATTAAAGCAAGCGCTTGGGGAAAAAGGAACGTGTGATTTCTTTTA
TGATACTGTTGGTGGTGAGCTTTTGGATAAAGTAATGACATTGATGACGAAAAACGG
TTTGATTTTGGTAGTTGGAGCAATTTCAGGTTTCAAGGATGTGACCAAGATGTATGTC
AAAAACTGGCCACAAGTAATTGCCAGTAGACTTACTATCAAGGGCTTTATTGTATCCG
ATTATCTTCACAAGGTAGAAGAAGCAAGGGTCAATCTTACCAAGTGGATTCAGGAAG
GAAAAATTCGGAACGATTCTAGTGTTTTAAACGTCATTGATTTGACCGAGGAGTCCAA
ATTTGCAGACATTCCCAAAGCTTGGGCGTTGATTCTTGACGAGAACAAGAAGCCAGG
AAAGGTATTGACGAAGCTTGCTGATCCGGAATAA

SDR

ATGGCAAAACAAGTATTTTTTATTGCTGGTGCTTCTCGTGGAATTGGTCTTAGTTTGGC
TACTTTATTAAGCAAAAATCCTGAAAATGTGGTTATTGCAACCGCTAGAAATCCTGCT
TCGGCTTCGGGTTTGCAAGAATTGAGCAAAGCAGACAACGTCCATGTTGTAACTTTGG
ACTTGAATAATGAAAAGACATATGAAACTGCCAAGGCAGATGTGTTGAAAATTTCAG

ATTCAATTGATGTATTCATTGTCAACGCAGGTATTAGTAACGCTCACGCCAAGGTTTT
GGACACTACAAAGGAAGAGTTCCTTAGTCACTTCACTACCAATACTCTTGGACCATTC
TTTTTGCTCCAAACCTTTTTGGGTTTGGTGAAAAAGGGTACTCTGAAAAAGGTGATTT
TCGTTTCTAGTGCTGCTGGTTCTGTAAGCGTGGAGCCTAGTTATGTTAGCTCCGCCTAC
GGTATTTCTAAGGCTGCACTCAACTATGGTGCTAGACAGATTGCTCGTGATCTCTCAG
AGGAGAATTTTATAGTGGTCCCTGTCCATCCAGGGTTGGTTGGTACTGATTTGCTTTTC
AATGCTACCGAGCCATTTCTCAAGAGTAACCCACATTTTAAGGATTTTCTTGGCCCTG
AAAACTACCTTACTCCTGATCAAAGTGCCAAGGGATTGGTGGCTTTGATTGAGAAGCT
TAAGAAGGAAGACTCCGGCAAGTTTTGGAACTACGATGGTACTGAGGTTCCG

XDH

ATGACTCCCAACCCATCTTTAGTGCTCAACAAGGTCAATGACATTACCTTCGAGACCC
TCGAAGCTCCTACGCTTCTGGAACCCAATGAGGTGATGGTCGAGGTGAAAAAAACCG
GTATTTGTGGAAGTGATATCCACTACTATTCCCATGGAAAAATCGGCGATTTTGTTCT
TACCCAGCCAATGGTTTTGGGTCACGAGTCCGCAGGTGTGGTTACGGCGGTGGGCCT
GAACGTGAAGTCGTTGAAGGTTGGAGATAGGGTGGCTATTGAGCCCGGAGTGCCTTC
GCGGTTTTCCGAAGAGTACAAAAGTGGGCACTATCAATTGTGTCCCAACATTGTATTT
GCGGCAACTCCAGACCCCAAGCACGGGTCTCCTAGTCCTCCTGGTACCCTCTGTAAGT
ATTACAAGTCGCCAGAAGATTTTCTTGTGAAACTTCCAGACTGCGTTTCCTTGGAGTT
GGGTGCGATGGTTGAACCACTTAGTGTCGGTGTCCATGGGTGTAAACAGGCCAAAGT
CACGTTTGGCGACGTGGTAGTGGTCTTTGGAGGAGGTCCGGTTGGTCTTTTGGCGGCA
GCAGCTGCAACCAAGTTTGGTGCTGCCAAAGTCATGGTGGTCGACGTCATTGACGAC
AAGTTAAAAATGGCTTTGGAAGTTGGTGTGGCCACCCATACGTTCAATTCCAAGTCTG
GAGGTGCTGACGAGCTTGTCAAGGAGCTTGGCGAACACCCAGATGTTGTAATTGAGT
GTACCGGTGCTGAAGTGTGTATCAATTTGGGCATTGAGAGCTTGAAAATGGGTGGTC
GTTTTGCTCAAGTAGGCAATGCCACTCGTCCCGTGAGCTTCCCAATCGTTGCATTTTC
GTCGCGTGAGTTGACTTTGTACGGCTCGTTCCGTTACGGATACAATGACTACAAAACC
AGTGTTGCAATCTTGGAACACAACTACCGTAATGGTCGTGAAAACGCAGCCATAGAC
TTTGAAAAGTTGATCACCCATCGATTCAAGTTTGAGGATGCAAAGAAGGCATACGAC
TACATTCGTGACGGAAACGTGGCAGTAAAAGTTATTATAGATGGTCCCGAGTAA
