# Supplementary figures and images for: A straightforward chemobiocatalytic route for one-pot valorization of glucose into 2,5-bis(hydroxymethyl)furan
Source: Bioresour Bioprocess. 2024 Apr 18;11(1):38. doi: 10.1186/s40643-024-00758-4 (PMC11026326; doi:10.1186/s40643-024-00758-4)

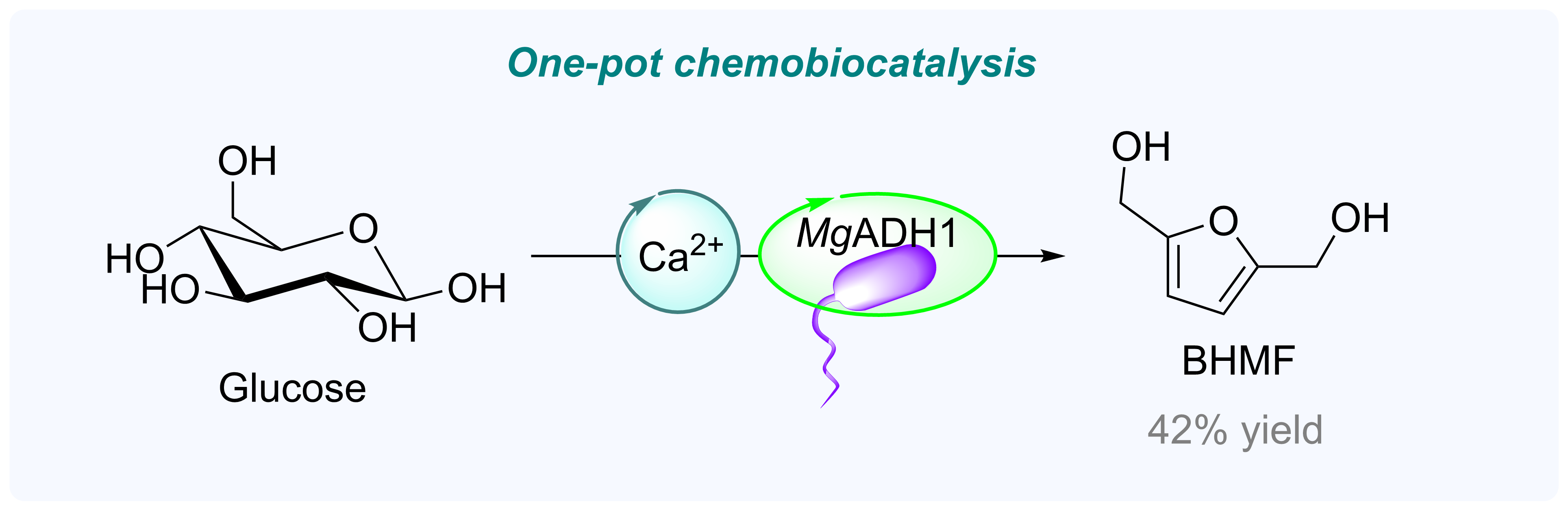

Supplement: Supplementary file 2 — Supplementary Material 2 [file 40643_2024_758_MOESM2_ESM.png]
